# COVID-19: Specific and Non-Specific Clinical Manifestations and Symptoms: The Current State of Knowledge

**DOI:** 10.3390/jcm9061753

**Published:** 2020-06-05

**Authors:** Jacek Baj, Hanna Karakuła-Juchnowicz, Grzegorz Teresiński, Grzegorz Buszewicz, Marzanna Ciesielka, Ryszard Sitarz, Alicja Forma, Kaja Karakuła, Wojciech Flieger, Piero Portincasa, Ryszard Maciejewski

**Affiliations:** 1 Chair and Department of Anatomy, Medical University of Lublin, 20-090 Lublin, Poland; ryszard.maciejewski@umlub.pl; 2 Chair and 1st Department of Psychiatry, Psychotherapy and Early Intervention, Medical University of Lublin, Gluska Street 1, 20-439 Lublin, Poland; karakula.hanna@gmail.com (H.K.-J.); r.sitarz@hotmail.com (R.S.); kaja.karakula@gmail.com (K.K.); 3 Department of Clinical Neuropsychiatry, Medical University of Lublin, Gluska Street 1, 20-439 Lublin, Poland; 4 Chair and Department of Forensic Medicine, Medical University of Lublin, 20-090 Lublin, Poland; grzegorzteresinski@umlub.pl (G.T.); g.buszewicz@umlub.pl (G.B.); ciesiela@umlub.pl (M.C.); aforma@onet.pl (A.F.); 5 Faculty of Medicine, Medical University of Lublin, Aleje Racławickie 1, 20-059 Lublin, Poland; wwoj24@wp.pl; 6 Clinica Medica A. Murri, Department of Biomedical Sciences and Human Oncology, University of Bari Aldo Moro Medical School, 70126 Bari, Italy; piero.portincasa@uniba.it

**Keywords:** Coronavirus disease 2019 (COVID-19), the severe acute respiratory syndrome coronavirus-2 (SARS-CoV-2), coronavirus, RNA, epidemic, pandemics, symptoms, outbreak, diagnosis, public health

## Abstract

Coronavirus disease 2019 (COVID-19), due to the severe acute respiratory syndrome coronavirus-2 (SARS-CoV-2), has become an epidemiological threat and a worldwide concern. SARS-CoV-2 has spread to 210 countries worldwide and more than 6,500,000 confirmed cases and 384,643 deaths have been reported, while the number of both confirmed and fatal cases is continually increasing. COVID-19 is a viral disease that can affect every age group—from infants to the elderly—resulting in a wide spectrum of various clinical manifestations. COVID-19 might present different degrees of severity—from mild or even asymptomatic carriers, even to fatal cases. The most common complications include pneumonia and acute respiratory distress syndrome. Fever, dry cough, muscle weakness, and chest pain are the most prevalent and typical symptoms of COVID-19. However, patients might also present atypical symptoms that can occur alone, which might indicate the possible SARS-CoV-2 infection. The aim of this paper is to review and summarize all of the findings regarding clinical manifestations of COVID-19 patients, which include respiratory, neurological, olfactory and gustatory, gastrointestinal, ophthalmic, dermatological, cardiac, and rheumatologic manifestations, as well as specific symptoms in pediatric patients.

## 1. Introduction

The first reported case of a severe acute respiratory syndrome coronavirus 2 (SARS-Cov-2) infection (Wuhan, Hubei Province, China), in December 2019, began the outbreak of a novel coronavirus disease (COVID-19), immediately becoming a huge global health concern. On 30 January 2020, COVID-19 was registered as the sixth Public Health Emergency of International Concern (PHEIC) by the World Health Organization (WHO), which was officially declared as a pandemic on 11 March 2020 [1,2]. Currently, there are approximately 6,500,000 confirmed cases of COVID-19 and more than 384,000 deaths, which were reported in more than 200 countries worldwide [3]. So far, the fatality rate due to COVID-19 varies from 1% to more than 7%, and the main causation remains a respiratory failure; however, the complete course of the disease is still not yet understood [4]. To compare, the mortality rates of the major previous epidemics—a severe acute respiratory syndrome (SARS) and the Middle East respiratory syndrome (MERS)—were estimated at 9.6% and up to 34.5%, respectively (Table 1) [5].

**Figure 1:**
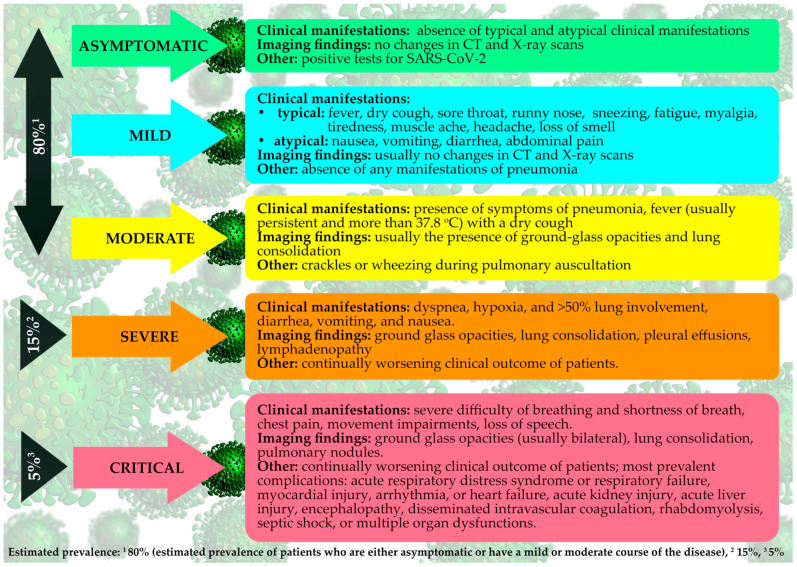
The severity of SARS-CoV-2 infection with typical characteristics.

Several risk factors are associated with the complications of COVID-19, and these include older age (>65), chronic respiratory diseases, cardiovascular diseases, hypertension, diabetes, and obesity. Acute respiratory distress syndrome (ARDS) is reported to be the most common complication [24,25]. Other severe or fatal complications include pneumonia, type I respiratory failure, sepsis, metabolic acidosis, septic shock, arrhythmia, acute cardiac injury, heart failure, acute kidney injury, bleeding, or hypoxic encephalopathy [26,27,28,29]. So far, males are registered to be infected with a higher prevalence compared to females and the reason is yet undiscovered [30,31]. COVID-19 lasts approximately 6 weeks and the duration, as well as the severity of the disease, depends primarily on the age and the immune system of an infected individual. Clinical manifestations can range from being mild to severe and patients can present as either symptomatic or asymptomatic, but a majority of COVID-19 cases are symptomatic with a moderate case-fatality rate (Figure 1) [25,32].

The majority of confirmed cases are aged 30–79 (86.6%), and the highest fatality rate is in a group of patients aged >80 years old [33]. Since an effective antiviral treatment is yet unavailable, clinicians worldwide make a significant effort to develop a vaccine and potential therapeutical drugs to minimize the fatal cases and alleviate the symptoms of COVID-19. Even though COVID-19 has a lower percentage of severity and mortality rates compared to SARS or MERS, it is much more transmissive and contagious and can affect everyone—from infants to the elderly—resulting in a wide spectrum of clinical manifestations [32,34,35].

## 2. General Symptoms

The majority of patients with COVID-19 present common symptoms that include fever, shortness of breath, cough (either with or without sputum), sore throat, nasal congestion, dizziness, chills, muscle ache, arthralgia, weakness, fatigue or myalgia, chest tightness, excessive mucus production with expectoration, hemoptysis, and dyspnea [36,37,38,39,40,41,42]. Even though fever is not the only initial clinical manifestation of SARS-CoV-2 infection, it is considered to be critical [43,44]. Fever, cough, and fatigue are the three most prevalent symptoms in COVID-19 patients [25,45]. Other less characteristic symptoms include headache, diarrhea, abdominal pain, vomiting, chest pain, rhinorrhoea, or pharyngalgia [46,47,48,49]. Approximately 90% of the patients present more than one symptom [50,51]. An approximate proportion of severe versus common cases of COVID-19 is estimated to 1:4 [52]. It is suggested that an early onset of shortness of breath constitutes a poor prognostic factor for patients. Among 81 fatal cases of patients from Wuhan, the most common cause of death was a respiratory failure (46.91%), followed by septic shock (19.75%), multiple organ failure (16.05%), and cardiac arrest (8.64%). Rarer death causes were acute coronary syndrome, malignant arrhythmia, or disseminated intravascular coagulation (DIC) [53]. Zhou et al. reported a case of a COVID-19 patient with a spontaneous pneumomediastinum and subcutaneous emphysema [54]. Clinical characteristics might differ between critically ill and non-critically ill patients [55,56].

### 2.1. Radiological Findings

The majority of patients show bilateral pneumonia and only a small percentage of COVID-19 patients show unilateral pneumonia. The most frequent computed tomography (CT) findings are bilateral patchy shadows and ground-glass opacities (GGO); multilobe involvement and focal lesions (patches, stripes, or nodules) are also very characteristic [57,58,59,60]. Less characteristic CT findings include centrilobular nodules, tree-in-bud sign, cystic change, pleural effusion, interstitial fibrosis, or lymphadenopathy. CT examinations show that lesions are more likely to be localized in the periphery than in the center of the lungs and the lesions are more patchy than oval [61,62]. Other CT findings include either pure GGO or GGO with reticular and/or interlobular septal thickening, GGO with consolidation, or pure consolidation [63,64]. Less common, but still characteristic, CT findings include ground-glass followed by irregular or halo sign, air bronchogram, bronchovascular bundle thickening, grid-form shadow, and hydrothorax [57]. Ground glass-like shadows, fibrous stripes, patchy shadow, and pleural thickening are observed both in common-type and severe or critical-type patients, independent to the severity of the COVID-19 course [65]. Single or multiple lobes of a single lung or both lungs (without a characteristic pattern) can be affected; interestingly, some of the studies showed that severe critical-type patients exhibit lesions primarily in the right lung [66].

### 2.2. Laboratory Findings

Generally, COVID-19 patients tend to have normal or decreased white blood cell counts, lymphopenia, or thrombocytopenia [67,68]. Zhang et al. showed that patients with high leukocyte count (>10 × 10^9^/L), higher neutrophil count (>7 × 10^9^/L), and lower lymphocyte count (<0.4 × 10^9^/L) are much more prone to severe COVID-19 pneumonia and composite endpoint (which was the admission to an intensive care unit, mechanical ventilation, or death) [69]. Besides, higher levels of C-reactive protein (>150 mg/L) and increased D-dimer levels (>1 mg/L) are also strongly associated with an increased risk of COVID-19 pneumonia and the composite endpoint. Additional laboratory indicators of increased risk are higher alanine aminotransferase (ALT) activity (>80 U/L), higher aspartate aminotransferase (AST) activity (>80 U/L), higher α—hydroxybutyrate dehydrogenase activity (>540 U/L), higher lactate dehydrogenase activity (>720 U/L), higher creatine kinase activity (>600 U/L), and lower total protein level (<60 g/L). So far, researchers have not observed a significant statistical association between altered platelet counts and creatinine levels with an increased risk of COVID-19-related pneumonia. As opposed to numerous studies, Zhang et al. showed that COVID-19 pneumonia and composite endpoint are associated with leukocytosis rather than leukopenia [69]. However, the abovementioned results differ among COVID-19 patients. Du et al. observed that the majority of COVID-19 patients (81.2%) had lowered eosinophil count and many patients had decreased hemoglobin and hematocrit, as well as decreased activated partial prothrombin time (APTT) and increased prothrombin time (PT) [53]. Among studied patients, 22.4% had increased procalcitonin levels and elevated levels of blood urea nitrogen or serum creatinine. It is still speculated whether eosinophilopenia might constitute a prognostic factor for COVID-19 patients. Some patients present progressive lymphopenia with a concurrent progressive neutrophilia [70]. However, among the most common reported laboratory findings, those of the highest prevalence include elevated levels of C-reactive protein and erythrocyte number, as well as increased myohemoglobin, liver enzymes, and muscle enzymes [25]. Additionally, patients with a severe course of COVID-19 usually have elevated D-dimer levels, increased procalcitonin, increased leukocyte number, and lymphocytopenia [29,71]. In some cases, lymphocytes and white blood cell levels might remain within physiological ranges. The decrease in the number of lymphocytes is generally observed in the CD4+ subpopulation. No significant changes are stated in the case of CD8+ and B cell subpopulations [72]. Further, interleukin 10 (IL-10), interleukin 6 (IL-6), interleukin 1 (IL-1), interleukin 2R (IL-2R), and tumor necrosis factor alpha (TNF-α) levels might exceed the upper limit in COVID-19 patients [73,74,75,76]. Chemokines, such as interferon gamma-induced protein 10 (IP-10) and monocyte chemoattractant protein 1 (MCP1), are also overexpressed during the course of COVID-19 [74].

## 3. Neurological Manifestations

Besides severe clinical manifestations, primarily of the respiratory system, SARS-CoV-2 presents neurotropic properties [75]. Autopsies have revealed the presence of SARS-CoV-2 nucleic acid in both cerebrospinal fluid and brain tissue of infected patients [77,78]. The entering of SARS-CoV-2 into the central nervous system is possible, either via hematogenous, lymphatic, synapse-connected, or retrograde neuronal routes [79,80]. Neuroinvasion of SARS-CoV-2 and the presence of neurological manifestations might be an explanation of the presence of neurological impairments without other typical symptoms of infection, especially in asymptomatic patients.

Neurological manifestations might occur in both symptomatic and asymptomatic patients. Neurologic manifestations are commonly described in COVID-19 patients, and these might involve the central nervous system, peripheral nervous system, and skeletal muscles [81]. Patients with a severe course of COVID-19 are more likely to develop neurological dysfunctions, among which acute cerebrovascular disease, conscious disturbance, and skeletal muscle injury are highly prevalent [82]. Helms et al. reported that patients with ARDS due to SARS-CoV-2 infection also presented encephalopathy, prominent agitation and confusion, acute ischemic strokes, or corticospinal tract signs [83]. Some patients manifest only neurological symptoms, including headache, languidness, malaise, cerebral hemorrhage, or cerebral infarction [84,85]. Cases of encephalitis, necrotizing hemorrhagic encephalopathy, strokes, epileptic seizures, or rhabdomyolysis associated with SARS-CoV-2 infection have also been described [86]. Similar to adults, neurological findings might appear in the case of infected infants, and this is so far reported as the observed upward gaze, dystonic bilateral leg extension, and alterations in a child’s responsiveness [87,88].

Duong et al. reported a case of a female with meningoencephalitis with concurrent hallucinations and disorientation without respiratory manifestations [89]. Other studies reported a possibility of the occurrence of the Miller Fisher syndrome, polyneuritis cranialis, or encephalopathy in COVID-19 patients [90,91]. Another neurological disease associated with COVID-19 is the Guillain–Barré Syndrome, reported as a neurological complication due to SARS-Cov-2 infection in several patients so far [92,93,94,95]. Detailed clinical, neurological, and electrophysiological examinations are crucial to assess neurological symptoms of COVID-19 patients. Additionally, the abovementioned examinations are highly important, since neurological manifestations could appear alone and might present as non-specific symptoms in patients infected by SARS-CoV-2.

## 4. Olfactory and Gustatory Dysfunctions

Isolated sudden-onset anosmia is reported to be the fourth the most common symptom of SARS-Cov-2 infection [96,97,98,99]. Further, patients who present sudden olfactory and/or gustatory dysfunctions irrespective of co-existing symptoms should be suspected of SARS-Cov-2 infection [100,101,102]. However, the pathogenesis of olfactory and gustatory dysfunctions in COVID-19 is still undiscovered. Approximately 79.7% of COVID-19 patients without nasal obstruction or rhinorrhea report hyposmia or anosmia [103]. The occurrence of fever is highly associated with the olfactory dysfunctions, and these may appear before, during, or after the general symptoms. The severity of olfactory dysfunctions differs from complete anosmia to severe, moderate, or mild microsmia or normosmia. Olfactory dysfunctions might persist even in up to 56% of patients who were reported as recovered from COVID-19 [103]. After the recovery, some of the olfactory dysfunctions might persist and gustatory dysfunctions might be resolved, and vice versa. There are also cases of complete losses of olfactory functions [104]. Anosmia might constitute the only symptom of COVID-19 [96,105,106]. The mean duration of smell and taste disorders due to SARS-Cov-2 is estimated at 7.5 days [107]. It was suggested that the presence of olfactory dysfunctions might constitute a potential (but limited) marker of SARS-Cov-2 infection [108,109]. Furthermore, loss of smell in COVID-19 patients might be associated with a milder clinical course of the disease [110].

## 5. Gastrointestinal and Hepatic Manifestations

A significant number of studies indicate that SARS-CoV-2 actively infects and replicates within the gastrointestinal tract, inducing digestive symptoms primarily via overexpression of viral receptor angiotensin-converting enzyme 2 (ACE2), found in gastrointestinal epithelial cells [111]. SARS-CoV-2 can be detected in the esophagus, stomach, duodenum, and rectum. It can also be found in the fecal samples [22,112,113,114]. Furthermore, it was shown that negative results from the nasopharyngeal swabs do not exclude viral infection, since the virus might be detected only in the rectal swabs [115]. The most common digestive symptoms in COVID-19 patients include nausea and/or vomiting, diarrhea, anorexia, or loss of appetite [116,117,118,119,120]. Rarer digestive symptoms include abdominal pain, abdominal distension, tenesmus, dysgeusia, gastrointestinal bleeding, or hematochezia [121,122,123,124]. Findings of Nobel et al. indicate that the presence of gastrointestinal dysfunctions might be associated with a more indolent form of COVID-19; such patients might present longer duration of the disease course [125]. Likewise, the severity of COVID-19 is associated with the more pronounced gastrointestinal manifestations. Gastrointestinal manifestations might constitute the only symptoms of SARS-CoV-2 infection without the impairments from the respiratory system or fever [126,127]. Further, COVID-19 patients might present gastrointestinal dysfunctions before the occurrence of other symptoms [128]. Despite prolonged prothrombin time and lowered monocyte counts, no significant differences were found in the complete blood count, electrolytes, or kidney functions in COVID-19 patients with gastrointestinal impairments [129].

Apart from the gastrointestinal manifestations, SARS-CoV-2 infection might involve liver impairments of a wide spectrum of a severity degree [125,130,131]. COVID-19 patients show increased levels of ALT and AST. Furthermore, serum bilirubin and gamma-glutamyl transferase (GGT) might also be elevated during the course of the disease [131,132,133,134,135]. Elevated levels of ALT and AST might be observed both in severe and non-severe cases of COVID-19 [136]. However, so far, it has been reported that liver injury due to SARS-CoV-2 infection occurs more prevalently in severe cases rather than mild cases of COVID-19. Lagana et al. reported a case of hepatitis associated with COVID-19 [137]. It must be mentioned that the pathological mechanism of liver injury is not yet understood; however, the possible mechanisms include direct viral infection of hepatocytes, drug hepatotoxicity, binding to cholangiocytes via ACE2 receptors, or immune-related injuries [132,138,139].

## 6. Ophthalmic Manifestations

Coronaviruses are capable of inducing a wide spectrum of ophthalmic manifestations, such as conjunctivitis, anterior uveitis, retinitis, or optic neuritis [140]. SARS-CoV-2 presents its ability of the ocular transmission, which might result in ocular manifestations; however, the prevalence of such incidents is extremely low [141,142]. Similarly, to other symptoms not related to the respiratory system, ophthalmic manifestations might appear as the first symptom without any other impairments. Besides, ocular impairments are rather more prevalent in patients with a severe course of the disease. The prevalence of ocular manifestations varies from 2% to 32% [143]. Compared to standard nasopharyngeal samples, the sensitivity of ocular swabs in SARS-CoV-2 detection is very low. It was reported that SARS-CoV-2 RNA can be detectable in ocular swabs days after being undetectable in the nasal swabs [144]. Ocular manifestations might occur relatively early during the COVID-19 course. It was reported that ophthalmic manifestations might be associated with the severity of the COVID-19 course [145]. Wu et al. showed that, among 38 infected patients, 12 presented ophthalmic manifestations, such as conjunctivitis, conjunctival hyperemia, chemosis, epiphora, or increased secretions [146]. Ocular manifestations primarily include the onset of conjunctivitis, keratoconjunctivitis, or ocular irritation symptoms [147,148,149,150,151]. Daruich et al. reported a case of an infected patient with unilateral eyelid edema and moderate conjunctival hyperemia [152]. Conjunctivitis can be the first symptom of SARS-Cov-2 infection [153].

## 7. Dermatological Manifestations

The SARS-CoV-2 infection has been reported to manifest in the form of cutaneous symptoms. The first report of skin involvement in COVID-19 patients was observed in the form of an erythematous rash, widespread urticaria, and chickenpox-like vesicles, especially occupying the trunk [154]. Mahé et al. reported a case of an infected patient with a distinctive skin rash [155]. It was suggested that, in some cases, skin lesions might constitute a late manifestation of COVID-19, especially in young healthy individuals, and might appear due to the immunological reactions [156]. Nevertheless, there are cases in which dermatological manifestations (e.g., acute urticaria with pyrexia) might occur first before other (more or less) characteristic symptoms [157]. COVID-19 patients might appear with the herpetiform lesions primarily located on the trunk; these lesions can be characterized by vesicles surrounded by erythematous halos with mild pruritus; vesicles might also form crusts [158,159,160]. Other researchers showed that skin lesions might appear as intensely pruritic, in form of a petechial rash, or even as an urticarial eruption [161,162,163]. Zulfiqar et al. reported a case of a COVID-19 patient with immune thrombocytopenic purpura [164]. Cutaneous manifestations associated with SARS-CoV-2 infection also include maculopapular exanthem, papulovesicular rash, urticaria, livedo reticularis lesions, or petechiae [165,166]. Since skin lesions in COVID-19 patients might be similar to those during the course of dengue, some patients might be easily misdiagnosed [167].

## 8. Cardiovascular Manifestations

Although cardiovascular diseases might significantly worsen the clinical outcome of COVID-19 patients, SARS-CoV-2 infection might also induce cardiac complications de novo [168,169]. Cardiac impairments might occur even without any symptoms or signs of pneumonia. The pathophysiological mechanisms probably involve ACE2 receptors, a cytokine storm induced by the imbalanced response between type 1 and 2 T-helper cells or strong interferon-mediated immunopathological events [43,170,171,172]. Further, atrial fibrillation, which is the most prevalent causation of arrhythmias, might be triggered by COVID-19-related hypoxia and the complications can persist even after pulmonary recovery [173]. Cardiac impairments might also be associated with pharmacological interventions (drugs currently used during COVID-19 treatment might prolong the QT interval or can be proarrhythmic). The most prevalent cardiovascular complication of COVID-19 is an acute myocardial injury (usually defined as an increase in cardiac troponin I above the 99th percentile upper reference limit), with a prevalence of 8%–12% [174,175,176]. An elevation of troponin levels is rather observed in a severe course of COVID-19, compared to mild or moderate courses. Increased levels of creatine kinase myocardial band (CK-MB), myohemoglobin, cardiac troponin I, and N-terminal pro-brain natriuretic peptide are associated with the severity of COVID-19 [177,178,179]. Other most prevalent complications include either brady- or tachyarrhythmias, with an estimated incidence of 16.7%, acute pericarditis, left ventricular dysfunctions, heart failure, cardiogenic shock, blood pressure abnormalities, or myocarditis [180,181,182,183,184]. It is very prevalent among COVID-19 patients that cardiac manifestations coexist with respiratory impairments [185]. Myocardial injury associated with SARS-CoV-2 infection impairs cardiac functions and induces ventricular tachyarrhythmias [186]. Inciardi et al. reported a case of a COVID-19 patient with an acute myopericarditis [187]. Zeng et al. described the first case of fulminant myocarditis as a COVID-19 complication [188]. The Kawasaki-like disease has been recently described as a post-infectious inflammatory syndrome that might constitute a complication of the COVID-19 disease, especially among pediatric patients [189,190]. Furthermore, acute pulmonary embolism and aortic thrombosis might be non-characteristic presentations in COVID-19 patients [191,192,193,194]. Similar to other viral types of pneumonia, patients infected by SARS-CoV-2 are at a higher risk of an acute pulmonary embolism. COVID-19 patients with pulmonary embolus have higher D-dimer levels compared to infected patients without pulmonary embolism [195,196]. Thus, a potential association between COVID-19 and pulmonary embolism should be taken into consideration, especially among patients with high D-dimer levels and without other clinical manifestations typical for COVID-19. Additionally, arterial and venous thromboembolic events are quite common cardiovascular manifestations among COVID-19 patients, which indicates a crucial role of COVID-19-associated coagulopathy [197,198,199]. A prominent elevation of D-dimer levels and higher levels of fibrin/fibrinogen degradation products are the most prevalent presentations of COVID-19-associated coagulopathy during the initial stages; altered coagulation parameters might be associated with poorer clinical outcomes of patients [200,201]. It was reported that antiphospholipid antibodies might be tested positive in COVID-19 patients and their presence might (rarely) induce thrombotic events [194]. The mortality rate of patients with cardiac injury due to SARS-CoV-2 infection is much higher compared to those without cardiovascular complications [202,203]. It was estimated that coagulation dysfunctions constitute the major cause of death in severely ill COVID-19 patients [204]. An endomyocardial biopsy of a COVID-19 patient with a cardiogenic shock showed that viral particles are not detected in myocytes specifically, but they can be detected in the interstitial cytopathic macrophages and their surroundings; myocytes were only characterized by focal myofibrillar lysis [205]. COVID-19 patients might also develop endothelial cell infection or endotheliitis, as it was observed that viral particles can be detected within endothelial cells, causing diffuse endothelial inflammation [206]. Cui et al. reported a case of an infected 55-days-old infant who, despite pneumonia, had liver injury and heart damage due to SARS-CoV-2 infection [207]. Hua et al. reported the first case of a COVID-19 patient with cardiac tamponade [208].

## 9. Rheumatology Symptoms

So far, data on the rheumatic manifestations in COVID-19 patients is still limited. It was reported that arthralgia might be an initial presentation of COVID-19 and this manifestation might be easily missed, especially in regions where the viral arthropod-borne disease is relatively common [209]. According to studies, lupus patients are more susceptible to SARS-CoV-2 infections and the course of COVID-19 might be very complicated in such patients [210]. Similarly, patients with rheumatoid arthritis are much more susceptible to SARS-CoV-2 compared to the general population [211].

## 10. Clinical Manifestations in Pediatric Patients

The amount of literature regarding clinical manifestations of COVID-19 in pediatric patients is continually increasing. Children usually present mild symptoms or might remain asymptomatic [212]. Pediatric patients with other comorbid diseases are much more vulnerable to SARS-CoV-2 infection and a more severe course of COVID-19 [213]. Generally, the majority of pediatric patients have mild symptoms, without fever or pneumonia, and the recovery time is estimated to be 1–2 weeks after the onset of the disease [214,215]. Such a mild course of COVID-19 in pediatric patients might be due to several reasons, including more effective immune responses in children, differences in the expression of the ACE2 receptor, or a simultaneous presence of other viruses in the respiratory tract of children, which might limit SARS-CoV-2 infection [216,217,218]. Children with COVID-19 present longer incubation periods compared to adults, estimated for 6.5 days in children and 5.4 days in adult patients [213]. The most common clinical manifestations in children include fever and cough; in some cases, additional symptoms, such as fatigue, myalgia, nasal congestion, runny nose, sneezing, sore throat, headache, vomiting, dizziness, or abdominal pain might be present [219,220,221,222,223,224]. There are also incidents of asymptomatic pediatric patients or those who only exhibit cough or diarrhea [225,226]. In some cases, infected infants of children might present typical symptoms, such as gastrointestinal manifestations, asthma, or shortness of breath alone [227]. Viner and Whittaker have reported that the Kawasaki-like disease might constitute a complication of the COVID-19 course, primarily in a population of pediatric patients [228]. Those atypical symptoms might significantly delay the diagnosis of COVID-19 in pediatric patients, potentially worsening their clinical outcomes [229]. Pediatric patients might have common clinical manifestations, such as fever or mild pneumonia, but a lot of reported patients have neither obvious symptoms nor abnormal CT findings [230,231]. However, the severe cases might progress to acute respiratory distress syndrome, septic shock, refractory metabolic acidosis, and coagulation dysfunctions [232,233].

The prevalence of SARS-CoV-2 infection among newborns and infants is very low but still possible and can manifest as asymptomatic, mild, or severe infection. So far, there is no evidence for the vertical transmission of the virus from mother to newborn; however, an infection is possible, primarily due to the close contact with an infected mother [234]. Newborns and infants infected by SARS-CoV-2 might present such manifestations as fever or mild upper respiratory symptoms alone but can also remain asymptomatic [235]. Generally, newborns and infants remain asymptomatic and present mild, non-specific symptoms, including cough, headache, runny nose, nasal congestion, expectoration, tachypnea, apnea, tachycardia, lethargy, vomiting, or diarrhea [236,237]. Gastrointestinal symptoms (diarrhea, food aversion, abdominal distension) are primarily observed among smaller infants [238]. Acute respiratory distress syndrome and temperature instability, as well as gastrointestinal and cardiovascular dysfunctions, are non-specific clinical features of infected (in particular preterm) infants and newborns [236]. Atypical clinical features of infants with COVID-19 also include neurological symptoms, such as axial hypotonia, drowsiness, or moaning sounds [88].

Regarding laboratory findings in pediatric patients, the white blood cell counts might be normal or decreased. Some patients might have leukopenia and only a small percentage present with lymphocytopenia. Severe or critical cases of pediatric COVID-19 patients might present elevated hepatic and muscular enzymes, as well as increased D-dimer levels [239,240]. Imaging findings usually present ground-glass opacities and segmental consolidation with surrounding halo signs, which is considered to be a typical sign in pediatric patients [239,241,242]. Regarding immunocompromised pediatric patients, and those with chronic diseases, current data does not suggest that such populations are at higher risk of severe infection [243].

## 11. Conclusions

The outbreak of SARS-CoV-2 infection, which started in Wuhan, China, in December 2019, has now become a global concern, being reported in more than 200 countries. The understanding of COVID-19, its diagnosis, transmission routes, molecular mechanisms of infection, prevention, and treatment strategies are rapidly evolving. Compared to previous infections of the severe acute respiratory syndrome-related coronavirus (SARS-CoV) or the Middle East respiratory syndrome-related coronavirus (MERS-CoV), SARS-CoV-2 is much more transmissive and dangerous and might affect nearly everyone, resulting in a wide spectrum of clinical manifestations. SARS-CoV-2 does not only affect the respiratory tract, resulting in pneumonia, but can affect the gastrointestinal, nervous, or cardiovascular systems. Less typical manifestations include dermatologic or ophthalmic manifestations. However, the pathomechanisms of the abovementioned manifestations are yet undiscovered in the majority of cases. The course of COVID-19 can be mild, moderate, severe, or critical; the number of asymptomatic carriers is also very high, worsening the epidemiological situation. Even though the long-term complications are unknown, pneumonia, acute respiratory failure, acute respiratory distress syndrome, acute liver or kidney injury, cardiac complications, septic shock, or coagulopathy are described, so far, as being the most prevalent. Some of the clinical manifestations that are not typical might appear first, predicting COVID-19; therefore, the knowledge about them is inherent.

## Figures and Tables

**Table 1:** Clinical features of severe acute respiratory syndrome coronavirus (SARS-CoV), Middle East respiratory syndrome coronavirus (MERS-CoV), and severe acute respiratory syndrome coronavirus-2 (SARS-CoV-2 infection).

	Severe Acute Respiratory Syndrome	Middle East Respiratory Syndrome	Coronavirus Disease 2019
	(SARS)	(MERS)	(COVID-19)
Disease-causing pathogen	SARS-CoV	MERS-CoV	SARS-CoV-2
First reported case	Southern China, 2002	Saudi Arabia, 2012	Wuhan, China, 2019
Symptoms	Fever, chills/rigor, myalgia, malaise, dry cough, shortness of breath (without upper respiratory tract symptoms), headache, dyspnea, excessive sputum production, sore throat, coryza, dizziness, nausea, vomiting, diarrhea [6]	Fever, cough, shortness of breath, malaise, chills, myalgia, headache, dyspnea, sore throat, nausea, vomiting, diarrhea, abdominal pain [7]	Fever, cough, shortness of breath, dyspnea, expectoration, muscle pain, fatigue, headache, sore throat, chest pain, chills, diarrhea, nausea, vomiting [8]
Imaging findings of the lungs	Ground-glass opacities Lung consolidation: focal, multifocal, or diffuse (primarily peripheral) Lung involvement: unilateral (two-thirds of patients) or bilateral Lesions: distributed within the lower lobes of the lungs [9]	Ground-glass opacities Lung consolidation Lung involvement: bilateral (80%) or unilateral (20%) Pleural effusion Intralobular septal thickening [10]	Ground-glass opacities: single or multiple focal Lung consolidation Patchy consolidative opacities Pulmonary nodules Interlobular septal thickening Bronchial wall thickening Lesions: usually bilateral, peripheral, and distributed within the lower lobes of the lungs [11,12]
Incubation period.	1–10 days [13]	2–14 days [14]	2–14 days [15]
Human-to-human transmission	Yes	Yes	Yes
Transmission routes	Close (droplets) contact with symptomatic patients [16] Contaminated surfaces [17]	Contact with infected camels or consumption of contaminated milk or meat [18] Limited human-to-human transmission (via droplets) [19]	Close (droplets) or distant (aerosol particles) contact with symptomatic or asymptomatic patients [20] Contaminated surfaces [21] Fecal transmission [22]
Mortality rate	9.6% [5]	34.5% [5]	2.3% [23]
